# Blunt Traumatic Hernia of Diaphragm With Late Presentation

**DOI:** 10.5812/atr.7593

**Published:** 2012-10-14

**Authors:** Abdolhossein Davoodabadi, Esmaeil Fakharian, Mahdi Mohammadzadeh, Esmaeil Abdorrahim Kashi, Azadeh Sadat Mirzadeh

**Affiliations:** 1 Trauma Research Center, Kashan University of Medical Sciences, Kashan, IR Iran; 2 Student Research Center Committee, Kashan University of Medical Sciences, Kashan, IR Iran

**Keywords:** Diaphragmatic Hernia, Trauma, Diaphragm

## Abstract

**Background:**

Diaphragmatic hernia after blunt trauma is an uncommon and often undiagnosed condition.

**Objectives:**

We aimed to review patients who presented with delayed blunt traumatic hernia of diaphragm.

**Patients and Methods:**

In this retrospective study, the medical records of six patients treated for blunt diaphragmatic hernias who were admitted to Kashan Shahid Beheshti hospital between June 2007 and June 2011 were analyzed.

**Results:**

Six patients with mean age of 41 years were included in the study. Male to female ratio was 2:1. Mean duration between trauma and admission to the hospital was 6.5 years (2 – 26 years). Five patients had left-sided diaphragmatic hernia. Chest X-ray was obtained from all patients which was diagnostic in 50 percent of the cases (n = 4). Additional diagnostic imaging with computerized tomography (CT) was used in six patients and upper gastrointestinal (GI) contrast study was performed in one patient. All patients underwent thoracotomy incision. Mesh repair was utilized in one patient. The mean hospitalization time was 14.1 days. There was one postoperative death (16.7%).

**Conclusions:**

Late presentation of blunt diaphragmatic hernia is an uncommon and challenging situation for the surgeon. Prompt diagnosis and treatment prevent serious morbidity and mortality associated with complications such as gangrene and perforation of herniated organ.

## 1. Background

Traumatic injury to diaphragm is an uncommon condition associated with severe blunt trauma which is usually missed in the early presentation to emergency (1, 2). Its incidence is approximately 2% in non-penetrating trauma. The increasing incidence of road traffic accidents has resulted in growing its incidence despite of advanced therapeutic modalities and awareness of medical staff (3, 4). The initial diagnosis of a blunt traumatic injury to the diaphragm is generally difficult because the early clinical and radiological findings are not clear. In acute circumstances, it is usually found on the table during exploratory laparotomy, although even then it may be missed (5). Nearly half of the patients remain asymptomatic and present with complications of the diseases several years after the onset of initial trauma (4, 6, 7). Undiagnosed patients with diaphragmatic injury may present with a diverse range of complaints from abdominal discomfort to diaphragmatic tear and visceral herniation secondary to sudden increase in the intra-abdominal pressure resulting in respiratory and gastrointestinal obstruction symptoms (8, 9). The incidence of diaphragmatic rupture after any kind of thoraco-abdominal trauma is reported between 0.8 and 5%, of which up to 30% present with a delay (10, 11). Less than 2.7% of the diaphragmatic injuries were detected before four months from the initial damage (8). Inaccurate interpretation of the imaging studies or intermittent and trivial symptoms of the hernia are main reasons for missed diagnosis (12, 13).

## 2. Objectives

Clinical presentation and management of 6 cases of delayed blunt traumatic hernia of diaphragm were discussed to highlight challenges in diagnosis and handling of complications.

## 3. Patients and Methods

Six patients with the diagnosis of delayed blunt traumatic hernia of diaphragm treated in Kashan Shahid Beheshti university hospital between June 2007 and June 2011 were enrolled to this study. The mechanism, duration of complaints, clinical presentation, site of injury, kind of visceral herniation, surgical repair, and outcome of the patients have been scrutinized. Diaphragmatic hernia repair and organ reduction were performed in all patients through thoracic approach.

## 4. Results

**Figure 1:**
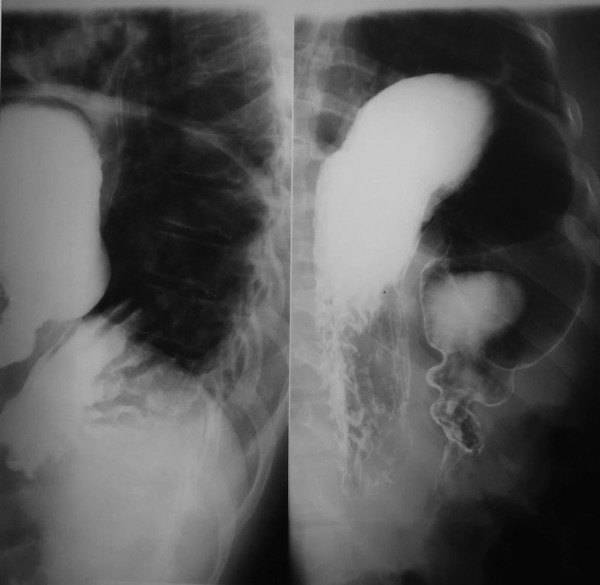
Upper Gastrointestinal (GI) Contrast Study of a Patient Presented With Intermittent Abdominal Pain, Vomiting, and Dyspnea, Especially after Heavy Meal, 26 Years from Car Accident

Mean age (± SD) of the patients was 41 years (± 15) (ranging from 25 to 67 years), with a male to female ratio of 2:1. The mean duration between the incident and admission to the hospital was 6.5 years (ranging from 2 to 26 years). All of the patients were victims of car accident. Five out of six patients were affected by left and one by right diaphragmatic hernias. All of the patients suffered from abdominal pain and two patients were admitted with the symptoms of intestinal obstruction. Four patients demonstrated respiratory symptoms, including dyspnea. Chest X-ray was performed for all patients as the initial diagnostic imaging work up. It was diagnostic in 3 patients and showed small bowel and colon loops in hemithorax, while it was recorded as atypical in the other 3 cases including 2 cases of lung empyema and diaphragmatic dome elevation in the last case. Both thoracic CT scan and barium study were diagnostic in all patients (Figure 1). Barium study (lateral view) shows that the stomach lies in left hemithorax (Figure 1). The most common herniated organ was stomach (n = 4). Transverse colon was herniated in 3 and liver in 2 patients. Two patients needed herniated organ resection due to micro-perforation and necrosis. The mean diameter of defect in the diaphragm after reduction was 11.1 cm (range: 6 - 16 cm). Primary repair was used in 5 patients and Prolene mesh in 1 patient. Mean hospitalization time was 14.1 days (± 3.4days). There was one postoperative death (16.7%), a 67 year-old man with history of car accident 2 years before operation who was admitted with abdominal pain, vomiting, and respiratory septic features (fever, tachycardia, tachypnea). The patient underwent left posterlateral thoracotomy with the diagnosis of thoracic empyma due to diaphragmatic herniation, and stomach torsion and gangrene. He died 2 days after the operation. Other patients were discharged without any complication (Table 1).

**Table 1.** Demographic Features, Clinical Findings, Diagnosis, and Management of Patients

	Variants
**Number , No.**	6
**Age, Mean**	41
**Gender , Male: Female**	4:2
**Time between operation and trauma, y**	6.5
**Hospitalization time ,d, Mean**	14.1
**Presenting symptoms**	
Abdomen pain	6
Dyspnea	3
Vomiting	3
Fever	2
**Localization, Left: Right**	5:1
**Diameter, cm, Mean**	11.6
**Chest X-ray**	
Diagnostic	3
Atypical	3
**Herniated organ**	
Stomach	4
Transverse colon	3
Omentum	2
Small bowel	1
Liver	2
Spleen	1

## 5. Discussion 

Early diagnosis of diaphragmatic injury is difficult and often delayed. It was detected in 1.3% of the patients in the acute phase with an Injury Severity Score (ISS) of > 15 upon admission to a trauma center. Approximately half of the cases had herniated tissues through the diaphragmatic tear (12). Sudden intra-abdominal pressure changes may produce diaphragmatic injury, either as a laceration or an avulsion (2). The rupture typically originates at the central tendineum or musculotendinous junction. Although the natural history of the diaphragmatic injury is unknown, some animal studies have shown that it might heal without development of hernia (14, 15). If diagnosis of the injury is missed after the first admission, patients may be asymptomatic or have intermittent chronic abdominal or respiratory symptoms due to increased intra-abdominal pressure including constipation, cough, or distension after heavy meal. Sudden increased intra-abdominal pressure may lead to herniation and obstruction of hollow organs complicating with strangulation and/or perforation of the herniated contents (16). Approximately 84% of all hernias were on the left side similar to the previous studies (16-18) possibly due to relative weakness of left hemi-diaphragm and protective effect of liver on right diaphragm. Diaphragmatic herniation with blunt trauma was more common in left hemi-diaphragm (12). In the present study, the only patient with right-sided diaphragmatic hernia was a 43-year-old female with a history of blunt trauma 5 years before admission. The patient was manifested by symptoms of chronic dyspnea and epigastric pain. During the operation, a diaphragmatic defect of 15 cm was detected through which right lobe of liver was herniated. Primary repair of diaphragm was performed to restore the defect. The mean interval between the trauma and operation was almost similar to that in previous studies. It was 6.5 years in our study, 5.9 years in Okan *et al*. (16) and 4.1 years in Reber *et al*. (17) studies. In Feliciano *et al*. (18) study, this time was 1 year, but it is noteworthy that their study was performed on diaphragmatic hernia after penetrating injuries. Except for a patient who showed chronic symptoms due to liver herniation through right hemi-diaphragm, other patients were admitted to the hospital with acute problems such acute abdomen and respiratory insufficiency that mandated urgent operative intervention. The initial chest X-ray was diagnostic only in 25% of patients where hemopneumothorax was visible in the thoracic cavity in acute setting of the injury (12). Similar to Reber *et al*. (17) and Worthy *et al*. (19), chest X-ray, as an initial imaging, was diagnostic in 50 percent of the patients. Although Okan *et al*. reported a higher percentage of diagnostic value for chest X-ray (up to 80%), they used the additional diagnostic tests because most of their patients had chronic and long–term complaints (16). In recent studies CT scan and MRI were used for additional imaging (16, 20, 21), while upper gastrointestinal (GI) contrast studies were used in earlier case reports (22). In the acute phase of diaphragmatic injury, CT scan is able to add 10% to the preoperative diagnostic value of chest X-ray (12). In delayed presentation, each of the CT scan and barium study was 100% diagnostic for diaphragmatic hernia detection in our study. Orkan *et al*., as well, reported that CT scan and MRI findings were 100% diagnostic in both penetrating and blunt mechanisms (16). Bodanapally *et al*. with the use of multi-detector CT scanning reported promising results in penetrating diaphragmatic injuries, with sensitivity, specificity, and accuracy rates of 87%, 72%, and 77%, respectively (23). Therefore, computed tomography is the diagnostic test of choice in suspected patients with diaphragmatic hernia. Similar to the previous case series (17, 18, 22), the preferred method of defect closure in our series was primary repair using interrupted non-absorbable sutures. Although in Orkan *et al*. study (16) the mean diameter of defect size and mean interval time between trauma and operation were less than those in our study. Orkan *et al*. used prosthetic material as a preferred method of defect closure. They explained that the mesh was used because defect size and the duration might cause loss of elasticity of the diaphragm. Stomach was the most common herniated organ in the present study. Since the most diaphragmatic injury occurred in left diaphragm, stomach could be considered as the most common herniated organ. Gastric volvulus associated with traumatic diaphragmatic hernia was relatively rare and made the diagnosis even more difficult (24-26). Stomach resection was performed in 2 patients due to micro-perforation and necrosis. During recent decades, advanced diagnostic modalities and postoperative management lead to decreasing mortality rates due to delayed diagnosis of diaphragmatic herniation from 25% to 10% (17, 22). However, the presence of strangulation with gangrene and perforation was related to increased morbidity and mortality. Mortality rate of undergone emergency repairs in this condition was up to 32% (27). There was one postoperative death in our study due to gastric torsion and gangrene (16.7%). Although most authors granted trans-abdominal approach to repair defects in acute injuries due to high incidence of associated abdominal injuries in this setting (12, 28), the definite treatment of blunt traumatic hernia of diaphragm was surgical repair with standard posterlateral thoracotomy and reduction of herniated organs into abdominal cavity first, and then repair of injured diaphragm. This approach allowed the surgeon to release strong adhesions between herniated viscera and pleura, and achieve complete clearance of pleural cavity and repair of diaphragmatic defect. While this approach may require additional laparotomy if small or large bowel has to be resected, nevertheless in our study, no patient needed to undergo laparotomy. Except for one patient who died after the operation due to critical situation (sepsis and stomach gangrene), others were discharged without any complication. In conclusion, delayed diagnosis and treatment of ruptured diaphragm are associated with increased rates of morbidity and mortality and necessitates early aggressive surgical intervention. Despite the fact that the incidence of diaphragmatic hernia is uncommon, it should be suspected in patients with gastro-intestinal complains and history of recent chest blunt trauma.
